# Validating the InterVA Model to Estimate the Burden of Mortality from Verbal Autopsy Data: A Population-Based Cross-Sectional Study

**DOI:** 10.1371/journal.pone.0073463

**Published:** 2013-09-13

**Authors:** Sebsibe Tadesse

**Affiliations:** Institute of Public Health, the University of Gondar, Gondar, Ethiopia; Swiss Tropical & Public Health Institute, Switzerland

## Abstract

**Background:**

In countries with incomplete or no vital registration systems, verbal autopsy data are often reviewed by physicians in order to assign the probable cause of death. But in addition to being time and energy consuming, the method is liable to produce inconsistent results. The aim of this study is to validate the InterVA model for estimating the burden of mortality from verbal autopsy data by using physician review as a reference standard.

**Methods and Findings:**

A population-based cross-sectional study was conducted from March to April, 2012. All adults aged ≥14 years and died between 01 January, 2010 and 15 February, 2012 were included in the study. The verbal autopsy interviews were reviewed by the InterVA model and physicians to estimate cause-specific mortality fractions. Cohen’s kappa statistic, sensitivity, specificity, positive predictive value, and negative predictive value were applied to compare the agreement between the InterVA model and the physician review. A total of 408 adult deaths were studied. There was a general similarity and just slight differences between the InterVA model and the physicians in assigning cause-specific mortality. Both approaches showed an overall agreement in 298 (73%) cases [kappa = 0.49, 95% CI: 0.37-0.60]. The observed sensitivities and specificities across causes of death categories varied from 13.3% to 81.9% and 77.7% to 99.5%, respectively.

**Conclusions:**

In understanding the burden of disease and setting health intervention priorities in areas that lack reliable vital registration systems, an accurate analysis of verbal autopsies is essential. Therefore, users should be aware of the suboptimal performance of the InterVA model. Similar validation studies need to be undertaken considering the limitation of the physician review as gold standard since physicians may misinterpret some of the verbal autopsy data and finally reach a wrong conclusion of the cause of death.

## Introduction

Developing countries generally lack consistent, timely, and reliable information on the level of cause-specific mortality fractions (CSMFs) in their populations [1]. Vital registration data are incomplete and contain only few physician-certified deaths [2]. Nevertheless, any meaningful health intervention policy and/or program must be informed by the cause of deaths (CODs) that are of the greatest importance locally. Verbal autopsy (VA) is a useful tool in such settings to establish the probable COD by interviewing a close caregiver or anyone who can provide witness to the death event [3].

There have been various attempts at validating physician reviews to interpret VA data [4-7]. However, the methodology is known to have several limitations. For example, physicians may differ systematically in their methods of interpreting VA data owing to their training, experience, and/or perceptions of local epidemiology, particularly when diagnostic criteria are not standardized amongst different physicians [8-10]. Hence, there may be inter and intra-reviewer variability among physicians that may lead to inconsistencies in COD data, hindering reliable temporal and spatial comparisons of mortality. They mostly use open history to reach a decision and may not account consistently for all indicators. They may also be influenced by their own biases, particularly for less obvious CODs for which decisions have to be made between equally likely diagnoses [11-15]. Moreover, the physician review process incurs remunerative costs, consumes time, and requires the involvement of physicians who are an already overstretched resource in low-income countries [8,16]. Furthermore, a large percentage of CODs assigned by VAs remain undetermined as physicians often disagree over a final COD classification, especially for deaths for which VAs were not successfully completed [10,17-21].

Different alternative methods to the physician review process for interpreting VA data have remained of limited use [22-24]. However, the use of the InterVA model to interpret VA data is a relatively new methodology that has just been explored to have the advantage of achieving the maximum spatial and temporal consistency [25-27]. Moreover, it requires minimal time and labor resources, especially in comparison with the physician review method. Also, it is freely available in the public domain, making it an ideal option for resource-constrained settings [28]. A new version of InterVA, InterVA-4, was launched in August 2012 along with the new WHO standards for VA. It was designed to incorporate the more specialized previous versions of the model for maternal and neonatal deaths, and to build on the experience from InterVA-3 and preceding models [29]. Further details of the approach used in InterVA models are available in a range of peer-reviewed publications which can be found under the “more info” section of its website (www.interva.net).

In order to design appropriate promotive, curative, and rehabilitative health services and to influence policy decisions, information on the burden of mortality at a population level is critically important. In response to this, the current study is designed to evaluate the performance of the InterVA-3 model as the physician alternative method for generating cause-specific mortality data from VAs in northern Ethiopia.

## Methods

A population-based cross-sectional study was conducted from March to April, 2012, in Dabat Health and Demographic Surveillance System site (HDSSs) hosted by the University of Gondar. The site is located in a district known as Dabat, northern Ethiopia, and has an estimated population of 46,165 living in 7 rural and 3 urban "kebeles" (the smallest administrative units in Ethiopia). The local communities largely depend on subsistence agriculture and information on vital events, like birth, death, and migration are collected quarterly [30].

### Study population and data collection

All adults aged ≥14 years and died between 01 January, 2010, and 15 February, 2012, in the area were included in the study. This period was preferred in order to obtain an adequate number of deaths without marked recall bias. It is believed that adult deaths were remembered very well.

Pre-tested and modified WHO and INDEPTH [31,32] designed VA questionnaire was used to collect the data. The VA questionnaire included an open narrative, medical history, and closed questions. The narrative section was used to record free explanations of the circumstances of death; the medical history sections were used to extract data from medical certificates, and the closed section dealt with specific signs, symptoms, and conditions leading to death. Three trained supervisors and nine data collectors who had rich experience in the job participated in the data collection processes. After obtaining an informed written consent, the data collectors interviewed a close relative, friend, or neighbor of the deceased person who witnessed the death. Considering the usual mourning period in the study area, data were collected after 45 days for recent death events.

The VA questionnaire was translated into “Amharic” (the local language) and back to English to maintain the consistency of the questions. The training of data collectors and supervisors emphasized issues, such as the selection of eligible respondents, approaching grieving respondents, time of interviews, and compiling narrative responses (ensuring that duration, frequency, severity, and the sequence of symptoms were mentioned). The principal investigator and the supervisors coordinated the interview process, made spot-checks, and reviewed the completed questionnaires daily to ensure the completeness and consistency of the data collected. They also conducted random quality checks by re-interviewing about 10% of the respondents. The VA questionnaire was pre-tested on 25 respondents who lived near Dabat and had similar characteristics with the study population in the district. Based on the pre-test results, the questionnaire was adjusted contextually. Data entry was carried out by the principal investigator and another independent data clerk and was then compared to check for any variations in results.

### Interpretation of VA data

The InterVA-3 model and the physician reviewed the same basic data from the VA questionnaire independently. That is, both methods utilized information collected in the open narrative and medical histories section together with the closed-ended section to assign the probable COD.

### Physician interpretation

Two independent physicians reviewed each VA questionnaire independently to assign a single COD based on ICD-10. The ICD-10 list had unique codes for diseases, signs, symptoms, abnormal findings, complaints, social circumstances, and external causes of injury [27]. The physicians met subsequently to reach consensus on cases where there were differences of opinion. If no physician consensus was reached after discussion, the COD was regarded as indeterminate. The physicians were trained in procedures on assigning COD and given details of the study area and study population. However, they were not given any special briefing on the probabilistic model so as not to encroach on their professional freedom. In spite of that however, their review process was closely monitored and that they be not direct beneficiaries of the research output was ensured.

### Interpretation of the InterVA model

The model relates a range of input indicators, such as age, sex, physical signs and symptoms, medical history, and the circumstances of death to likely CODs using Bayesian probabilities [27]. The model results in up to three likely causes per case when possible; each associated with a quantified likelihood. To assign an estimate of the overall certainty for that patient, the model gives the average likelihood for a maximum of three CODs [28]. In this study, a high prevalence of Malaria and HIV/AIDS were used as basic epidemiological parameters for the model as their prevalence varies from place to place. Data were entered case-by-case into Microsoft visual FoxPro window of the InterVA version 3.2 to assign the possible COD responsible for the death of each individual.

### Comparison of the InterVA model with the physician

The most probable CODs assigned by the model were considered to facilitate comparison with the single CODs which were assigned by the physician. All CODs in both methods were re-categorized into 9 main groups for two reasons. The first reason was to have meaningfully comparable COD categories between both methods. Second, it was more important that the model and the physician arrive at a broad agreement in identifying COD groups with the greatest public health importance at a population level, rather than individual level causes. The list of the 9 main categories used in this study was: pulmonary tuberculosis (TB), HIV/AIDS-related deaths, diabetes, other infectious diseases, digestive diseases, cardiovascular problems, maternity-related deaths, other non-communicable diseases, and injuries/accidents.

Then deaths were aggregated case-by-case to their respective COD categories to determine the CSMFs at the community level by using both the InterVA model and the physician review. Cohen’s kappa statistic, sensitivity, specificity, positive predictive value (PPV), and negative predictive value (NPV) were applied to compare the agreement between the InterVA model and the physician review.

### Ethical considerations

The study protocol was reviewed and approved by the Institutional Ethical Review Board of the University of Gondar. Then, informed written consent was obtained from the study participants who were close relatives, friends, or neighbors of the deceased after explaining the purpose and the procedures of the study. Confidentiality was granted for information collected from each study participant. Study participants found sick at the time of data collection were referred to the nearest health institution for medical treatment. There was no remuneration for family.

Finally, for the purpose of completeness, findings of the previous study on population characteristics, interpretations of VA data, and others which were specific to pulmonary TB were included in this study [17]. The current and the previous studies were conducted in the same study area and study period using the same data source.

## Results

### Characteristics of the study population

A total of 408 VA interviews were successfully completed and reviewed by both the InterVA model and the physicians. Of the deceased, 222 (54.4%) were females. Two hundred eighty-one (68.9%) of the deceased were 50 and above years of age. Most of the deceased, 325 (79.7%), and 298 (90.0%), were married and farmers, respectively. As far as education is concerned, 308 (73.0%) of them were illiterate. The Majority, 306 (75.0%), of the deceased were rural dwellers.

### Physician interpretation

Out of the 408 deaths, 329 (80.6%), were successfully assigned a single cause at the first attempt by two physicians. After holding consensus meetings, the physicians successfully assigned a single COD to 61 (15%) more cases. Therefore, on the whole, physicians assigned a single COD to 390 (95.6%) cases. No consensus was reached on 18 (4.4%) cases which were coded as "indeterminate" by the physicians.

### Interpretation of the InterVA model

The InterVA-3 model assigned a single COD to 356 (87.3%) cases, two CODs to 52 (12.8%) cases, and three causes to 5 (1.2%) cases. In 10 (2.5%) cases, the InterVA model assigned the COD as "indeterminate". The probabilistic model assigned the likely CODs to all the VAs with a certainty of 75.0% and standard deviation of 2.8.

### Comparison of the InterVA model with the physician

**Figure 1:**
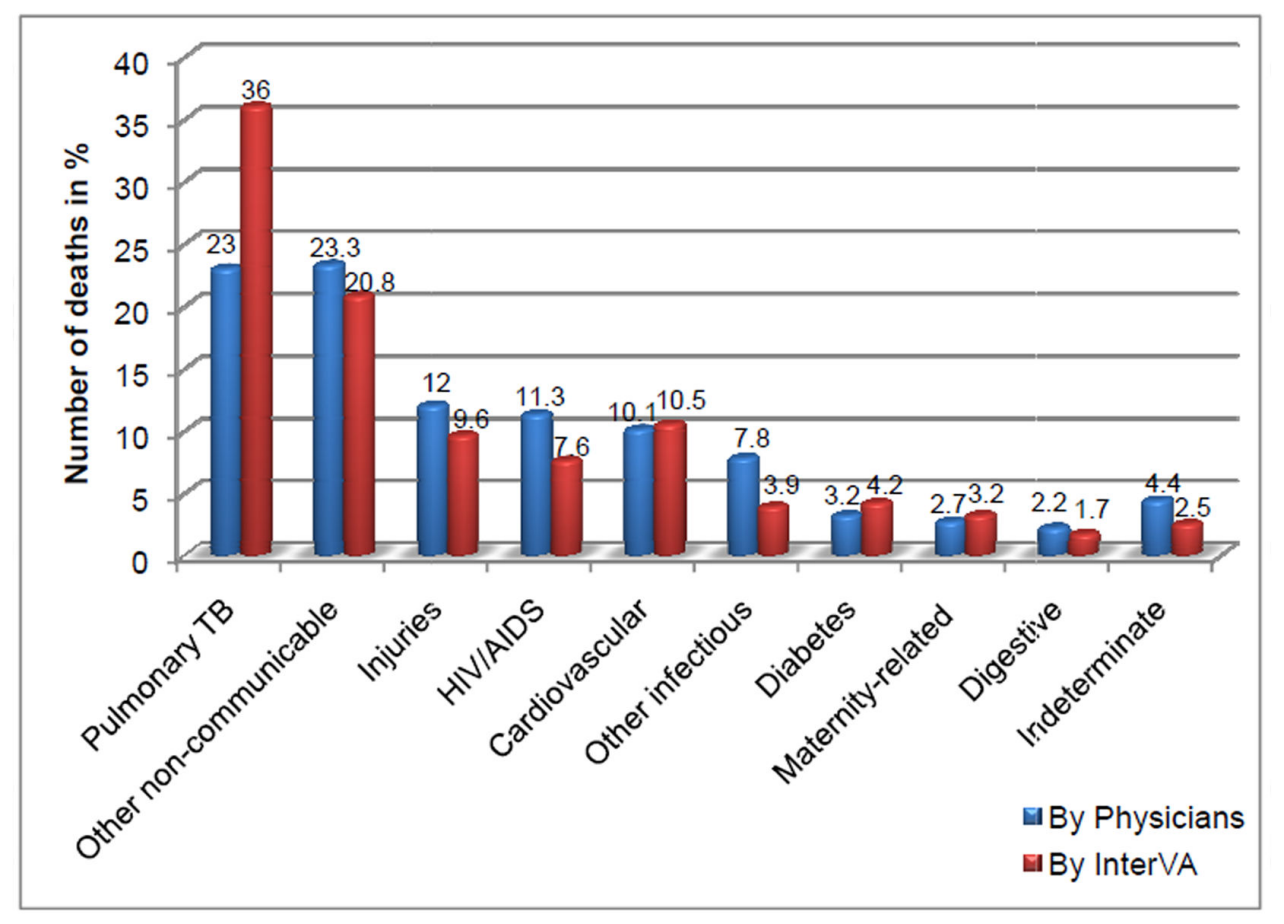
The CSMFs derived from physician review and InterVA model interpretation.

There was a general similarity and just slight differences between the InterVA model and the physicians in assigning cause-specific mortality. Out of all deaths in this population, two major groups of causes, pulmonary TB and other non-communicable diseases, accounted for about half of the overall mortality, as determined by both approaches. It is noteworthy that the InterVA model assigned significantly more CODs to pulmonary TB [147 (36.0%)] compared to physicians [94 (23.0%)]. On the other hand, physicians identified HIV/AIDS as a COD more frequently [46 (11.3%)] than the model [31 (7.6%)], (Figure 1).

A direct comparison of the CODs assigned by the physicians to the first CODs assigned by the InterVA model showed that there was an overall agreement in 298 (73%) cases [kappa = 0.49, 95% CI: 0.37-0.60]. The observed level of agreement across the COD categories varied from kappa value of 0.17 to 0.83. A poor level of agreement was observed only for digestive disease, (Table 1).

**Table 1:** Case-by-case agreement between the InterVA model and the physicians in establishing the CODs in northern Ethiopia from 01 January 2010–15 February 2012.

**COD Categories**	**Kappa(95%CI**)
1. Injuries/accidents	0.83 (0.76, 0.90)
2. Maternity-related death	0.76 (0.60, 0.92)
3. Diabetes	0.52 (0.36, 0.68)
4. Pulmonary TB	0.50 (0.40, 0.60)
5. Other infectious diseases	0.46 (0.34, 0.58)
6. Cardiovascular	0.42 (0.33, 0.51)
7. HIV/AIDS-related death	0.40 (0.30, 0.50)
8. Other non-communicable diseases	0.31 (0.23, 0.40)
9. Digestive diseases	0.17 (0.01, 0.33)

The results for sensitivities, specificities, PPV, and NPV of the InterVA model in comparison with the physicians were presented for COD categories. The observed sensitivities and specificities across the COD categories varied from 13.3% to 81.9% and 77.7% to 99.5%, respectively, (Table 2).

**Table 2:** Validating the InterVA model against the physician in diagnosing COD categories in northern Ethiopia from 01 January 2010–15 February 2012.

**Five COD categories**	**Sensitivity (%**)** (%**)	**PPV (%**)	**Specificity (%**)	**NPV**
1. Pulmonary TB	81.9	52.4	77.7	93.5
2. Injuries/accidents	76.5	95.1	99.4	96.7
3. Maternity-related death	72.7	80.0	99.5	99.2
4. Diabetes	61.5	47.0	97.7	98.7
5. Cardiovascular	45.8	51.2	94.2	92.9
6. Other infectious diseases	41.4	60.0	97.9	95.6
7. Other non-communicable diseases	36.4	49.1	91.2	86.0
8. HIV/AIDS-related death	33.3	51.6	95.8	91.5
9. Digestive diseases	13.3	25.0	98.5	96.8

## Discussion

In this study, the probabilistic InterVA model found out very similar results with the physicians for assigning cause-specific mortalities from VA data at the population level. This was true with other studies [7,25,33-35]. The frequencies of mortalities revealed were consistent with the existing knowledge on the burden of diseases among an underdeveloped population in sub-Saharan Africa [36-39], indicating good performance of the InterVA model for generating cause-specific mortality data from VA.

The high discordance observed between the two approaches in assigning pulmonary TB and HIV/AIDS as CODs in this study is supported by other investigations [7,20,25]. This might be due to a great deal of overlap between both disease conditions in terms of clinical symptoms and signs. Furthermore, the re-emergence of pulmonary TB in several countries of the world is spurred by the HIV/AIDS pandemic. This underlies the high level of interconnectedness between both diseases. Moreover, from a public health perspective, control and prevention of either disease cannot be considered without regard to the other [40-42]. So what is critical is that the collective burden of both diseases in any population is clear, and the InterVA model demonstrated this as successfully as the physicians did.

The observed level of agreement for varied COD categories indicated a fairly good diagnostic performance of the InterVA model. A nearly similar level of overall agreement was observed between the InterVA model and physicians [kappa = 0.42 (0.37-0.48)] in a validation study conducted in Kenya [6]. This confirmed the temporal and spatial consistency of the InterVA model for establishing cause-specific mortalities. However, a higher level of agreement between both approaches was observed in studies which utilized data collected by a demographic surveillance system [19,43]. The reason for the poor level of agreement observed for digestive diseases could be the overlapping nature of the clinical signs and symptoms with other diseases, especially HIV/AIDS.

Studies indicated that the validation of VA is considered to have an acceptable level of diagnostic accuracy at the population level, if sensitivity and specificity are at least 50% and 90%, respectively [22]. In this study, the observed sensitivity values were above 60% for pulmonary TB, injuries/accidents, maternity-related death and diabetes. However, lower sensitivity values were observed for deaths related with cardiovascular diseases, other infectious and non-communicable diseases, HIV/AIDS and digestive diseases. In previous studies, the sensitivity value for cardiovascular-related COD varied from 25% to 87% [4-6,22,23,44-46]. The observed specificity values were good, except for pulmonary TB. These criteria of validation (sensitivity at least 50% and specificity at least 90%) are not uniformly regarded as acceptable [47] because low sensitivity and specificity does not necessarily imply low level of accuracy, or relatively high sensitivity and specificity may result in serious misclassification errors. In the case of low sensitivity and specificity, the false positives and false negatives may counterbalance, and may not affect the VA accuracy [44,48].

Literatures reveal that there are robust validation metrics other than Cohen’s kappa, sensitivity and specificity to assess how well a VA method estimates CSMFs. These are: chance-corrected concordance, absolute CSMF errors, relative CSMFs error, and CSMF accuracy [5,7,20-22,45,49-54]. An average chance-corrected concordance across causes is recommended for assessing how well a method does at individual COD assignment. This metric is insensitive to the CSMF composition of the test sets and corrects for the degree to which a method will get the cause correct due strictly to chance. For the evaluation of CSMF estimation, CSMF accuracy is proposed. CSMF accuracy is defined as one minus the sum of all absolute CSMF errors across causes divided by the maximum total error. It is scaled from zero to one and can generalize a method’s CSMF estimation capability, regardless of the number of causes where a value of one means no error in the predicted CSMFs, and a value of zero means the method is equivalent to the worst possible method of assigning cause fractions [54].

A validation study of VA often faces the question as to how to obtain a true gold standard. Several studies have used CODs based on hospital diagnoses as the gold standard [5,6,9,44,50]. However, hospital diagnoses have limitations as gold standard since the composition and distribution of hospital CODs may not be representative of deaths occurring in the community. Moreover, in resource-constrained healthcare settings, hospital diagnoses which are often unavailable are of low quality when available and are limited by inadequate clinical data and record keeping. Furthermore, the ability to recognize, recall, and report signs of illnesses may be different among hospital users and nonhospital users. In this study, physician review was used as a reference standard to examine InterVA. The use of physician review was the only alternative source of COD assessment for this study population. This choice however has limitations. Physicians are influenced by their experience, perception, and interpretation of local epidemiology that may lead to inconsistencies in COD data, hindering reliable temporal and spatial comparisons of COD. Moreover, they often use open history to reach decisions and may not account consistently for all the indicators. They may also be influenced by their own biases, particularly for less obvious CODs for which decisions had to be made between equally likely diagnoses. These inherent limitations of physicians could lead them to misinterpret some of the VA data and finally reach a wrong conclusion of COD. Previous VA literature has also suggested that the physician review is not a robust method to interpret VA data [55]. Therefore, considering a physician review as a true gold standard to validate the InterVA model could influence the true diagnostic accuracy of the InterVA model.

The current study can only provide evidence on how the COD estimates derived from InterVA compared to those ascertained by the physician review. It cannot infer the performance of the InterVA compared to other existing methods which have been shown to perform better than InterVA previously. Studies proved that other automated options such as the Tariff Method, Simplified Symptom Pattern, Random Forests, and Machine Learning for the analysis of VA data have validated performance equal to or better than physician review [53,56-59]. Given the widespread use of VA for understanding the burden of disease and setting health intervention priorities in areas that lack reliable vital registrations systems, accurate analysis of VAs is essential. Therefore, users should be aware of the suboptimal performance of the InterVA in relation to other methods.

The other possible limitation of this study could be the cross-sectional study design which might not be appropriate for establishing cause-specific mortalities accurately. Using data from a well-established longitudinal demographic surveillance system may reduce the effect of recall biases associated with a long recall period. The absence of some variables in the WHO adult VA questionnaire is a factor challenging the diagnostic accuracy of the InterVA model. The model does not employ open-ended questions which are more relevant in a society with poor knowledge of symptoms of certain diseases and where more local terms may be used in this case. Even though the data collectors in this study had a long experience in field data collection processes, none of them had academic expertise in medical diagnosis of diseases which might adversely affect the quality of the data collected. This could in turn result in misleading interpretations by both the InterVA model and the physicians and finally lead to a wrong conclusion of COD. This study applied 9 broad COD categories which clearly increased the possibility that the two methods would agree. Therefore, an additional sensitivity analysis should be performed to see the impact of any change in the COD categories chosen on the level of agreement between the two methods. Another limitation could be the relatively small sample size of the study which might also contribute to the underestimation of the sensitivity and specificity values. Besides, the indeterminate probability of the COD would decrease if more than two physicians reviewed the data, but this was not done due to the inadequacy of the budget.

## Conclusions

In understanding the burden of disease and setting health intervention priorities in areas that lack reliable vital registrations systems, an accurate analysis of VAs is essential. Therefore, users should be aware of the suboptimal performance of the InterVA model. Similar validation studies need to be undertaken considering the limitation of the physician review as gold standard since physicians may misinterpret some of the VA data and finally reach a wrong conclusion of the COD.

## Supporting Information

File S1 **Detailed descriptions for the calculations of kappa, sensitivity, specificity, positive and negative predictive values were available for further reference.**
(DOCX)
