# Supplementary material for: Validating the InterVA Model to Estimate the Burden of Mortality from Verbal Autopsy Data: A Population-Based Cross-Sectional Study
Source: PLoS One. 2013 Sep 13;8(9):e73463. doi: 10.1371/journal.pone.0073463 (PMC3772846; doi:10.1371/journal.pone.0073463)
Supplement: File S1 — Detailed descriptions for the calculations of kappa, sensitivity, specificity, positive and negative predictive values were available for further reference. (DOCX) [file pone.0073463.s001.docx]

**Additional File S1 Kappa, sensitivity, specificity, positive predictive and negative predictive values were calculated from the 2x2 contingency table by using the following formulae.**

1. **Kappa = po—pe** where, **po** = a+d , **pe** = (a+b * a+c) + (c+d * b+d) ,N = a+b+c+d

**1—pe** N N N N N

**Po** = observed agreement, **pe** = expected agreement

1. **Sensitivity = a**

**a+c**

1. **Specificity = d**

**b+d**

1. **Predictive value positive = a**

**a+b**

1. **PrKedictive value negative = d**

**d+c**

Where, **a** = true positive, **b** = false positive, **c** = false negative, **d** = true negative

- **Cause of death category**

1. **Pulmonary TB**

Sensitivity = 81.9

Specificity =77.7

Predictive value positive =52.4

Predictive value negative =93.5

Kappa = 0.50

| **InterVA model** | **Physician review** | | **Total** |
| --- | --- | --- | --- |
|  | **Yes** | **No** |  |
| **Yes** | 77 | 70 | 147 |
| **No** | 17 | 244 | 261 |
| **Total** | 94 | 314 | 408 |

1. **Injuries**

Sensitivity = 76.5

Specificity = 99.4

Predictive value positive = 95.1

Predictive value negative = 96.7

Kappa = 0.83

| **InterVA model** | **Physician review** | | **Total** |
| --- | --- | --- | --- |
|  | **Yes** | **No** |  |
| **Yes** | 39 | 2 | 41 |
| **No** | 12 | 355 | 367 |
| **Total** | 51 | 357 | 408 |

1. **Maternity-related death**

Sensitivity = 72.7

Specificity = 99.5

Predictive value positive = 80.0

Predictive value negative = 99.2

Kappa = 0.76

| **InterVA model** | **Physician review** | | **Total** |
| --- | --- | --- | --- |
|  | **Yes** | **No** |  |
| **Yes** | 8 | 2 | 10 |
| **No** | 3 | 395 | 398 |
| **Total** | 11 | 397 | 408 |

1. **Diabetes**

Sensitivity = 61.5

Specificity = 97.7

Predictive value positive = 47.0

Predictive value negative = 98.7

Kappa = 0.52

| **InterVA model** | **Physician review** | | **Total** |
| --- | --- | --- | --- |
|  | **Yes** | **No** |  |
| **Yes** | 8 | 9 | 17 |
| **No** | 5 | 386 | 391 |
| **Total** | 13 | 395 | 408 |

1. **Cardiovascular diseases**

Sensitivity = 45.8

Specificity = 94.2

Predictive value positive = 51.2

Predictive value negative = 92.9

Kappa = 0.42

| **InterVA model** | **Physician review** | | **Total** |
| --- | --- | --- | --- |
|  | **Yes** | **No** |  |
| **Yes** | 22 | 21 | 43 |
| **No** | 26 | 339 | 365 |
| **Total** | 48 | 360 | 408 |

1. **Other infectious diseases**

Sensitivity = 41.4

Specificity = 97.9

Predictive value positive = 60.0

Predictive value negative = 95.6

Kappa = 0.46

| **InterVA model** | **Physician review** | | **Total** |
| --- | --- | --- | --- |
|  | **Yes** | **No** |  |
| **Yes** | 12 | 8 | 20 |
| **No** | 17 | 371 | 388 |
| **Total** | 29 | 379 | 408 |

1. **Other non-communicable diseases**

Sensitivity = 36.4

Specificity = 91.2

Predictive value positive = 49.1

Predictive value negative = 86.0

Kappa = 0.31

| **InterVA model** | **Physician review** | | **Total** |
| --- | --- | --- | --- |
|  | **Yes** | **No** |  |
| **Yes** | 28 | 29 | 57 |
| **No** | 49 | 302 | 351 |
| **Total** | 77 | 331 | 408 |

1. **HIV/AIDS-related death**

Sensitivity = 33.3

Specificity = 95.8

Predictive value positive = 51.6

Predictive value negative = 91.5

Kappa = 0.40

| **InterVA model** | **Physician review** | | **Total** |
| --- | --- | --- | --- |
|  | **Yes** | **No** |  |
| **Yes** | 20 | 11 | 31 |
| **No** | 32 | 345 | 377 |
| **Total** | 52 | 356 | 408 |

1. **Digestive diseases**

Sensitivity = 13.3

Specificity = 98.5

Predictive value positive = 25.0

Predictive value negative = 96.8

Kappa = 0.17

| **InterVA model** | **Physician review** | | **Total** |
| --- | --- | --- | --- |
|  | **Yes** | **No** |  |
| **Yes** | 2 | 6 | 8 |
| **No** | 13 | 387 | 400 |
| **Total** | 15 | 393 | 408 |
